# Genome-wide characterization of the *Triplophysa dalaica slc4* gene family and expression profiles in response to salinity changes

**DOI:** 10.1186/s12864-022-09057-8

**Published:** 2022-12-13

**Authors:** Chuanjiang Zhou, Bo Hu, Yongtao Tang, Xin Chen, Zhigang Ma, Qiqi Ding, Guoxing Nie

**Affiliations:** 1 College of Life Sciences, Henan Normal University, Xinxiang, 453007 People’s Republic of China; 2 College of Fisheries, Engineering Technology Research Center of Henan Province for Aquatic Animal Cultivation, Henan Normal University, Xinxiang, Henan 453007 People’s Republic of China

**Keywords:** *Triplophysa dalaica*, Solute carrier 4 family, Evolution, Gene expression

## Abstract

**Background:**

The solute carrier 4 (SLC4) gene family is involved in a variety of physiological processes in organisms and is essential for maintaining acid-base balance in the body. The *slc4* genes have been extensively studied in mammals, and they play important roles in intracellular and extracellular pH regulation and in the secretion and uptake of HCO_3_^−^ and other ions (Na^+^ and Cl^−^) between transepithelial cells in different tissues. This study identified and characterized the entire *slc4* gene family of *Triplophysa dalaica*.

**Results:**

Fifteen *slc4* genes were identified in the whole genome of *Triplophysa dalaica* in this study, including five copies of Na^+^-independent Cl^−^/HCO_3_^−^ transporters, eight members of Na^+^-dependent HCO_3_^−^ transporters, and two genes coding Na^+^-coupled borate transporters. The chromosomal location information, isoelectric points, and molecular weights of the 15 *slc4* genes were analyzed. The results for gene structure, domain analysis, and phylogenetic relationships of this gene family showed that the *slc4* genes (except for *slc4a9*, which is missing in teleosts) are significantly expanded in teleosts compared to higher vertebrates. This phenomenon suggests that the *slc4* gene family played an important role in the transition from aquatic to terrestrial animals. RT-PCR results showed that different *slc4* genes showed diversified expression patterns in the tissues of *T. dalaica*. For osmotic pressure regulating organs, *slc4a1b, slc4a4b, slc4a7*, and *slc4a11a* were highly expressed in gills. In the kidney, *slc4a1a, slc4a3*, and *slc4a10b* were highly expressed, suggesting that the *slc4* genes play a specific role in the salinity adaptation of *T. dalaica*. Our study has deciphered the biological roles of the *slc4* genes in maintaining ionic and acid-base homeostasis in teleost fishes and provides a foundation for future exploration of the highly differentiated gene family in *Triplophysa*.

**Conclusions:**

The results are relevant for the breeding of alkali-tolerant varieties in saline-alkali areas for aquaculture. Our findings have important implications for the adaptation process of freshwater species to saline-alkali water.

**Supplementary Information:**

The online version contains supplementary material available at 10.1186/s12864-022-09057-8.

## Background

The solute carrier family 4 (SLC4), also known as the bicarbonate transporters family, consists of 10 transporters in mammals and is essential for maintaining acid-base balance in the body [1]. The genes are divided into three categories according to their transport ions. The first category comprises the Na^+^-independent Cl^−^/HCO_3_^−^ exchangers (AEs) AE1 (SLC4A1), AE2 (SLC4A2), and AE3 (SLC4A3) that perform Cl^−^ and HCO_3_^−^ electroneutral transmembrane exchange. The second type is Na^+^-dependent HCO_3_^−^ transporters (NCBT) comprising the two electrically neutral Na^+^/HCO_3_^−^ cotransporters NBCe1 (SLC4A4) and NBCe2 (SLC4A5), two electrically neutral Na^+^/HCO_3_^−^ cotransporters NBCn1 (SLC4A7) and NBCn2 (SLC4A10), and an electrically neutral Na^+^-driven Cl^−^/HCO_3_^−^ exchanger NDCBE (SLC4A8). At present, the Na^+^-dependence of AE4 (SLC4A9) remains controversial. Some researchers believe that the product of SLC4A9 undergoes Na^+^-independent Cl^−^/HCO_3_^−^ exchange [2–5]. However, Park observed in a preliminary study that human AE4 mediates Na^+^-dependent HCO_3_^−^ transport rather than Na^+^-independent Cl^−^/HCO_3_^−^ exchange. The third class is Na^+^-coupled borate transporters (BTR1, SLC4A11) [6, 7].

At present, research on the *slc4* genes in fish is limited, primarily focused on a single or several members of the *slc4* gene family. There is increasing evidence to suggest that the *slc4* genes are closely related to the acid-base regulation and ion regulation of fishes [8, 9]. For example, it has been suggested that *slc4a1* and *slc4a4* play key roles in the ion-regulatory pathway of zebrafish gills [10, 11] suggested that *slc4a1*, *slc4a2*, and *slc4a4* are involved in the alkali adaptation process in the gill and kidney of *Leuciscus waleckii* [12]. observed a salinity-dependent expression pattern of the *slc4a4* genes in the foregut of *Sparus aurata* L. Studies have also shown that the *slc4a4* genes are significantly induced in *Takifugu obscurus* [13] and *Oreochromis mossambicus* [14] during seawater domestication. In 2020, researchers conducted a systematic study of the *slc4* gene family in *Lateolabrax maculatus* and found that the expression patterns of the *slc4* genes in the gill tissue were different in response to alkalinity stress and salinity changes in terms of the degree of differential expression. Understanding how these closely related proteins perform different functions is therefore one of the major challenges and opportunities for studying transporter families.

*T. dalaica* inhabits rivers with relatively gentle water flow. It is small in size and has strong adaptability. It is widely distributed in northern China in the tributaries of the Yellow River and some artesian water bodies such as Dali Lake in Inner Mongolia. Dali Lake is located in the internal flow basin of eastern Inner Mongolia (116°39′24″E, 43°22′43″N); its alkalinity can be as high as 53.57 mmol/l, and its pH can reach 9.62 [15]. After long-term natural environment domestication, the fish composition of Dali Lake at present consists of fish with high salt tolerance such as *Leuciscus waleckii*, *Carassius auratus* Linnaeus, *Pseudorasbora parva*, and *T. dalaica* [16]. suggested that *T. dalaica* has the strongest resistance to salinity and alkalinity and can survive in different water quality conditions. Therefore, *T. dalaica* is an ideal model species for studying the mechanism of adaptation to highly saline environments. At present, there are few domestic studies on the salinity adaptability of loach [17, 18]. In this study, the *slc4* gene family was identified; phylogenetic and gene structure analyses were performed; and the expression of *slc4* genes in different tissues of *T. dalaica* in freshwater and saline-alkali water habitats was examined. The expression patterns of the family members involved in salinity adaptation were explored, providing a new basis for studying the adaptation mechanisms of *slc4* genes in *T. dalaica* to saline environments and in addition promoting the aquaculture of suitable breeding varieties in saline-alkali areas.

## Results

### *Slc4* genes prediction and sequence features

A total of 15 *slc4* genes were identified in *T. dalaica*, and these were divided into three categories according to existing studies [7, 19]. There were five genes corresponding to Na^+^-independent Cl^−^/HCO_3_^−^ exchangers (*slc4a1a, slc4a1b, slc4a2a, slc4a2b,* and *slc4a3*), eight members of Na^+^-dependent HCO_3_^−^ transporters (Na^+^/ HCO_3_^−^ cotransporters, *slc4a4a, slc4a4b, slc4a5a, slc4a5b, slc4a7, slc4a8, slc4a10a,* and *slc4a10b*), and two genes belonging to Na^+^-coupled borate transporters (Na^+^/Borate cotransporter, *slc4a11a* and *slc4a11b*). Table 1 summarizes the basic characteristics of members of this gene family. The subcellular localization prediction results showed that the *slc4* genes were all localized on the cell membrane, further confirming that the members of this family are all integral membrane proteins. The predicted coding sequences of the *slc4* family ranged from 797 (*slc4a11b*) to 1230 (*slc4a3*) amino acids; their relative molecular weights ranged from 89.34 kDa to 139.56 kDa. The predicted pI ranged from 5.57 to 8.42. The number of exons was 15–26 (Table 1).

**Table 1:** Summary of characteristics of *slc4* gene family members in the *T. dalaica* genome

Classification	Gene name	CDS length (bp)	No. of exons	Isoelectric point	Molecular weight (kDa)	Predicted protein length (amino acid)	Subcellular localization
Cl^−^/HCO_3_^−^exchanger	*slc4a1a*	2721	20	5.57	101.6	906	Cell membrane
	*slc4a1b*	2565	16	6.57	95.45	854	Cell membrane
	*slc4a2a*	3672	22	5.99	137.04	1223	Cell membrane
	*slc4a2b*	3663	22	5.84	136.45	1220	Cell membrane
	*slc4a3*	3693	20	6.14	139.56	1230	Cell membrane
Na^+^/HCO_3_^−^cotransporter	*slc4a4a*	3219	23	6.11	120.59	1072	Cell membrane
	*slc4a4b*	3234	26	6.39	120.51	1077	Cell membrane
	*slc4a5a*	3399	25	6.11	127.78	1132	Cell membrane
	*slc4a5b*	3393	25	6.63	127.68	1130	Cell membrane
	*slc4a7*	3372	25	5.97	126.17	1123	Cell membrane
	*slc4a8*	3315	25	6.06	124.28	1104	Cell membrane
	*slc4a10a*	3345	25	5.84	125.4	1114	Cell membrane
	*slc4a10b*	3252	24	5.85	121.6	1083	Cell membrane
Na^+^/Borate cotransporter	*slc4a11a*	2511	15	6	94.37	836	Cell membrane
	*slc4a11b*	2394	19	8.42	89.34	797	Cell membrane

### Gene structure analysis of *slc4* genes

**Fig. 1:**
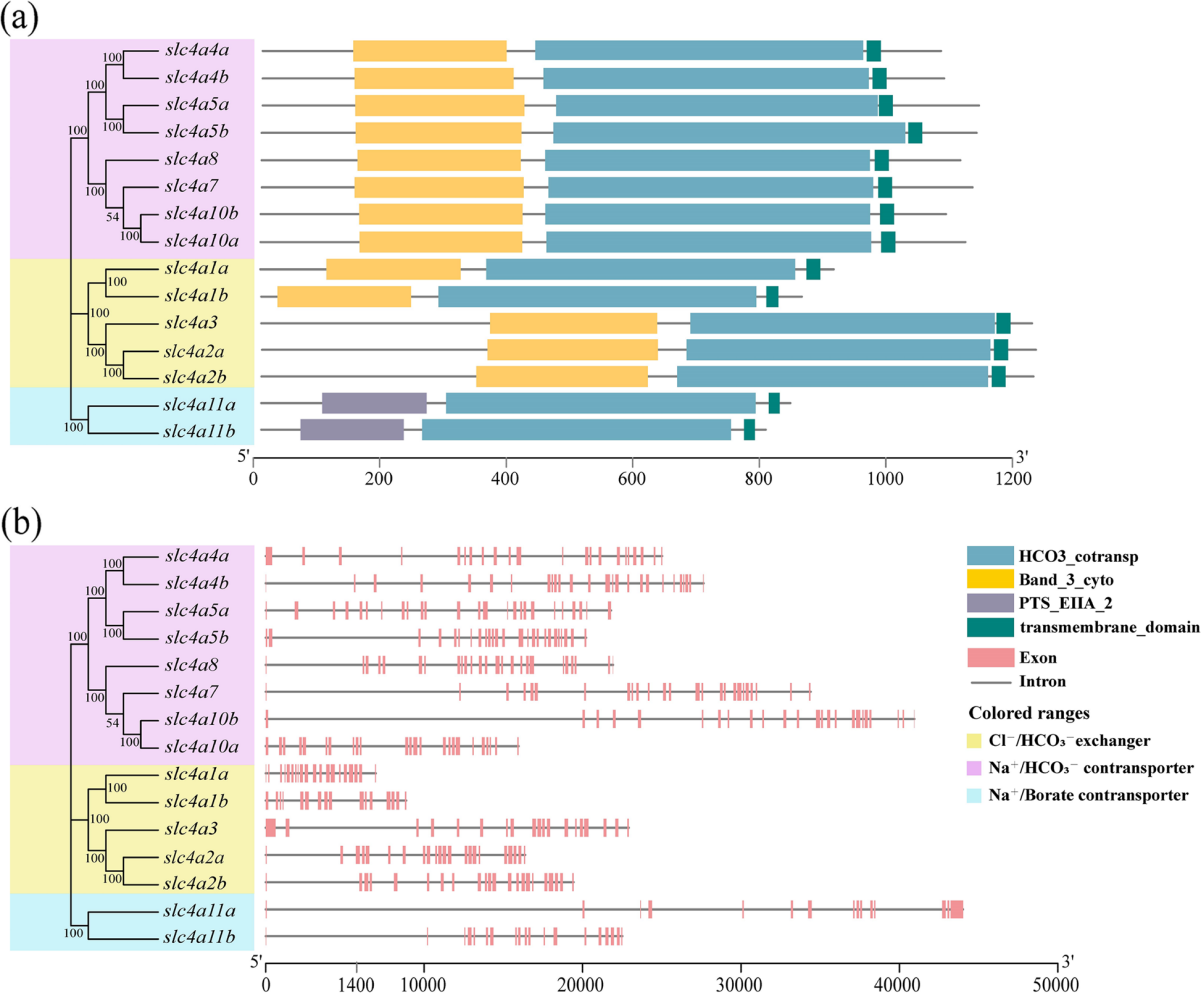
Gene structure analysis of *slc4* genes in *Triplophysa dalaica*. The phylogenetic tree on the left was constructed based on the neighbor-joining method in MEGA 7 with a bootstrap value of 1000, and the branch colors represent different groups. **a** The domains of the *slc4* gene family. **b** The pink box and black line on the right represent exons and introns, respectively

To compare the structural diversity of the *slc4* gene family, its domains and exon-intron structures were visualized using TBtools (Fig. 1). The results showed that most members of the Cl^−^/HCO_3_^−^ exchangers possessed 20–22 exons, except for *slc4a1b* that had 16 exons. Among the members of the Na^+^/HCO_3_^−^ transporters, *slc4a5a, slc4a5b, slc4a7, slc4a8*, and *slc4a10a* consisted of 25 exons. There were 23, 26, and 24 exons in *slc4a4a, slc4a4b*, and *slc4a10b*, respectively. There were 15 and 19 exons, respectively, in the Na^+^-coupled boronate transporters *slc4a11a* and *slc4a11b*. In addition, three functional domains were predicted in the *slc4* gene family, namely Band_3_cyto, HCO3_cotransp, and PTS_EIIA_2. HCO3_cotransp, located at the C-terminus of all *slc4* genes, indicating that it is highly conserved. Band_3_cyto exists in Cl^−^/HCO_3_^−^ exchanger and Na^+^/HCO_3_^−^ cotransporter. Band_3_cyto is a cytoplasmic domain that associates with the cell membrane cytoskeleton proteins to stabilize the membrane skeleton. PTS_EIIA_2 was only present in *slc4a11a* and *slc4a11b.* Overall, the predicted results of the exon-intron structures and domains of the 15 *slc4* genes were consistent with those of the phylogenetic tree.

### Motif analysis of *slc4* genes

**Fig. 2:**
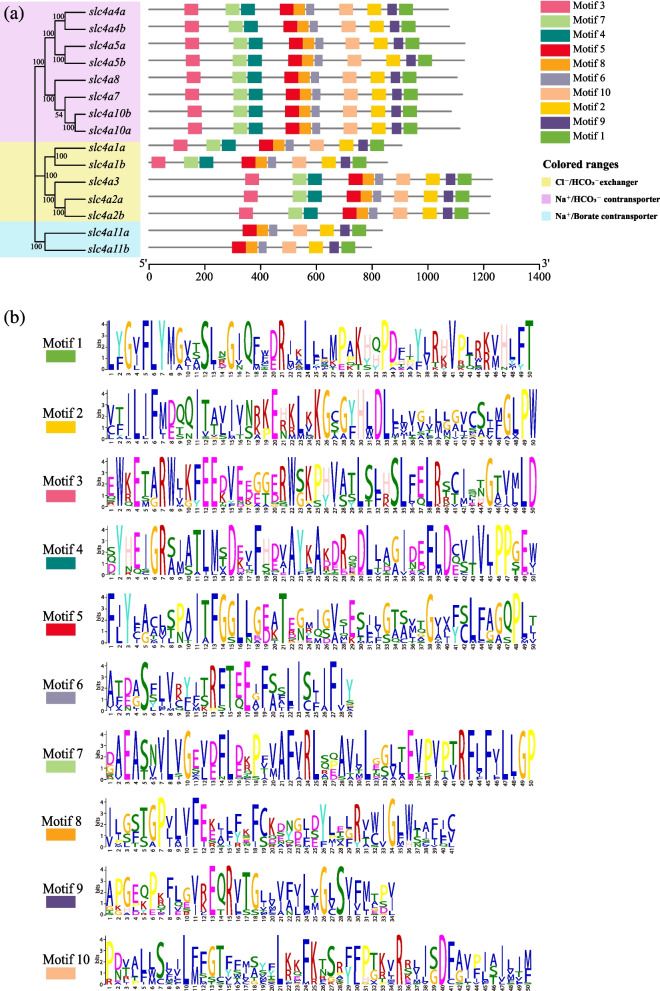
Conserved motif analysis of the *slc4* gene family of *Triplophysa dalaica*. **a** Phylogenetic relationships and structure of conserved protein motifs in the *slc4* proteins from *T. dalaica*. The phylogenetic tree same as Fig. 1. All motifs were identified from the MEME database, and different colored blocks represent different motifs. **b** Motifs of *T. dalaica slc4* proteins

The *T. dalaica slc4* genes were searched through the MEME database, and a total of 10 conserved motifs were identified (Fig. 2). Each motif ranged from 29 to 50 amino acids in length. Motif1–Motif10 are included in the members of Cl^−^/HCO_3_^−^ exchangers and Na^+^/HCO_3_^−^ cotransporters. Two genes in the Na^+^/Borate cotransporter group (*slc4a11a, slc4a11b*) contained only seven conserved motifs, excluding Motif3, Motif4 and Motif7. Motifs 1, 2, 5, 6, 8, 9, and 10 were shared by 15 *slc4* genes, suggesting that they are located in the main functional domain of *slc4* genes, namely, the HCO_3__cotransp domain that is responsible for the transport of anions. Overall, genes from the same transporter group showed similar motif characteristics, and the distribution of motifs was consistent with the results of the phylogenetic analysis.

### Phylogenetic analysis and copy number analysis of *slc4* genes

In this study, 202 amino acid sequences of the *slc4* genes of 15 representative vertebrates (4 of which belong to the genus *Triplophysa*) were selected to construct a phylogenetic tree. Table 2 summarizes the copy numbers of *slc4* gene members in *T. dalaica* and other vertebrates. It can be seen that the selected species range from higher vertebrates to teleost fishes, and the number of *slc4* genes varies from 10 to 15. All *slc4* genes in selected in higher vertebrates (human, mouse, *Xenopus laevis*) are single-copy, and duplicate *slc4* gene copies are present in common teleost genomes. The above results indicate that the *slc4* gene family expanded significantly during the evolution of teleost fishes. Furthermore, the *slc4a9* gene is not found in all teleosts.

**Table 2:** Comparison of copy numbers of *slc4* genes between *T. dalaica* and other species

Species name	Common name	*slc4a1*	*slc4a2*	*slc4a3*	*slc4a4*	*slc4a5*	*slc4a7*	*slc4a8*	*slc4a9*	*slc4a10*	*slc4a11*
*Homo sapiens*	human	1	1	1	1	1	1	1	1	1	1
*Mus musculus*	mouse	1	1	1	1	1	1	1	1	1	1
*Xenopus laevis*	clawed frog	1	1	1	1	1	1	1	1	1	1
*Danio rerio*	zebrafish	2	2	1	2	2	2	1	0	2	1
*Gasterosteus aculeatus*	stickleback	2	2	1	2	1	1	1	0	2	2
*Dicentrarchus labrax*	European sea bass	2	2	1	2	2	1	1	0	2	2
*Ictalurus punctatus*	channel catfish	2	2	1	2	2	2	1	0	2	1
*Larimichthys crocea*	large yellow croaker	2	2	1	2	2	2	1	0	2	1
*Oreochromis niloticus*	Nile tilapia	1	2	1	2	2	2	1	0	2	2
*Oryzias latipes*	Japanese medaka	2	2	1	2	2	2	1	0	2	1
*Takifugu rubripes*	fugu	2	1	1	1	2	1	1	0	2	2
*Triplophysa siluroides*	/	2	2	1	2	2	1	1	0	2	2
*Triplophysa tibetana*	/	2	2	1	2	2	1	1	0	2	2
*Triplophysa bleekeri*	/	2	2	1	2	2	1	1	0	1	1
*Triplophysa dalaica*	/	2	2	1	2	2	1	1	0	2	2

**Fig. 3:**
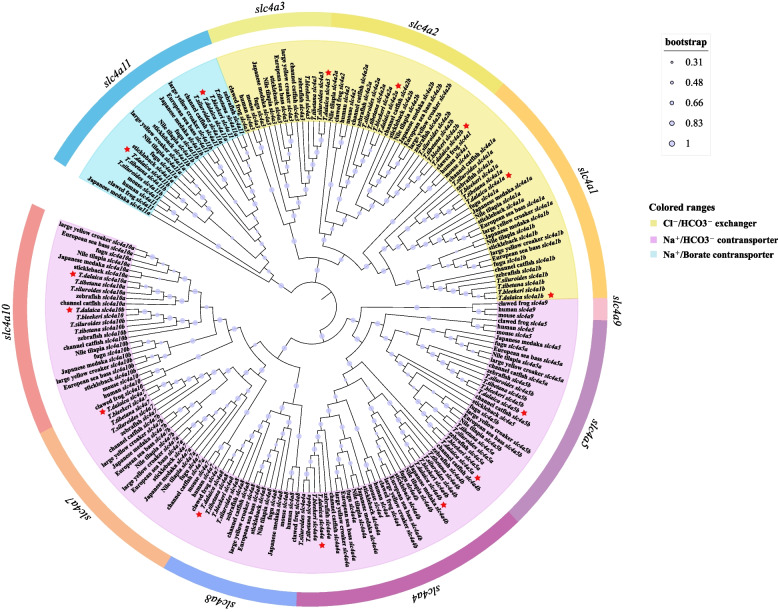
Phylogenetic analysis of the *slc4* genes of *T. dalaica* and selected species. The *slc4* genes of *T. dalaica* are marked with red stars. Branch shading represents different groups, and different colors on the outer ring represent different genes

Phylogenetic analysis can provide a basis for the annotation of *slc4* genes in *T. dalaica*. According to the gene clustering results shown in Fig. 3, all *slc4* genes were divided into three categories: Cl^−^/HCO_3_^−^ exchangers, Na^+^/HCO_3_^−^ cotransporters, and Na^+^/Borate cotransporters. Genes within these three categories were further divided into 10 separate clades. The *slc4* genes (red stars) of the *T. dalaica* were in the corresponding clade with other species and obtained a high support rate (boostrap value). The *slc4* genes of a total of four fish species in the *Triplophysa* genus were clustered with orthologous genes of other species, especially zebrafish, indicating that they have been highly conserved in the evolutionary process. The clustering results were consistent with those of previous studies [20, 21].

### *Slc4* gene collinearity analysis and chromosomal location

**Fig. 4:**
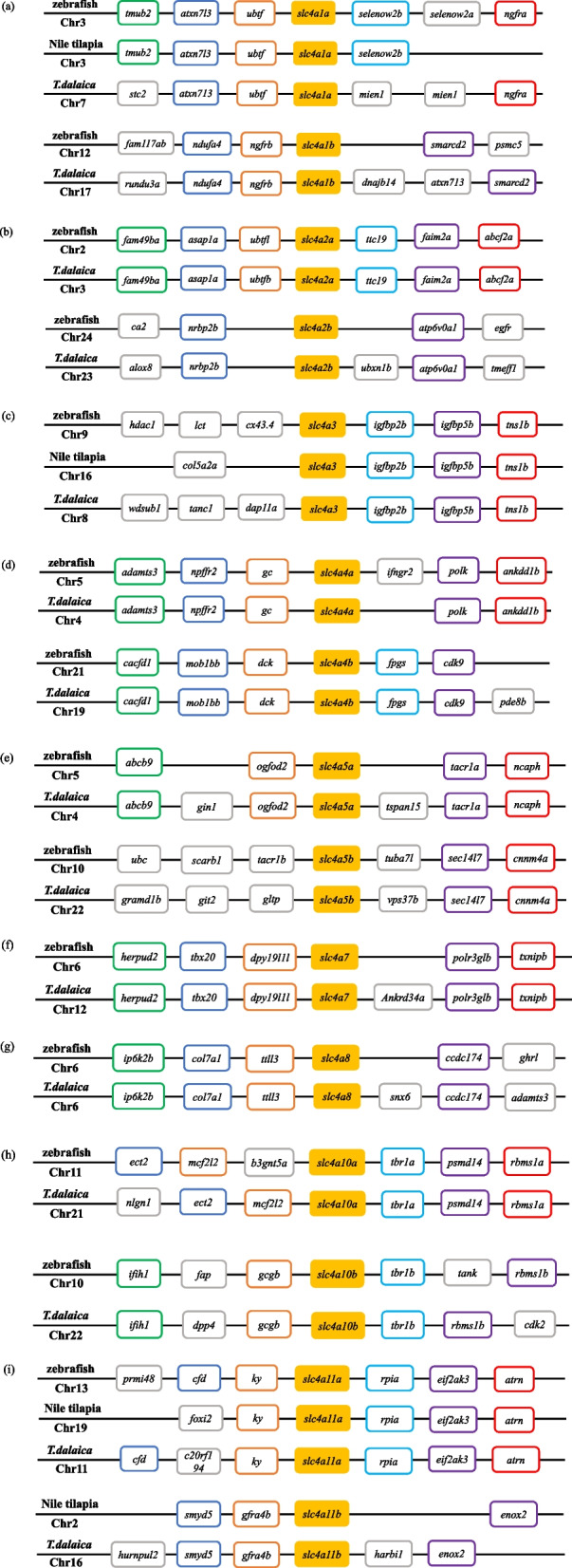
Syntenic analysis of *slc4* genes in zebrafish, Nile tilapia, and *T. dalaica.* a–i: *slc4a1*–*slc4a11* genes. The *slc4* genes are shown as filled colored boxes

Phylogenetic relationships supported the annotation of three single-copy genes (*slc4a3, slc4a7,* and *slc4a8*). To confirm the evolutionary relationship of the *slc4* genes with multiple copies (*slc4a1, slc4a2, slc4a5, slc4a7, slc4a10*, and *slc4a11*) and to provide more evidence for annotation, the adjacent genetic regions of the *slc4* genes were analyzed for collinearity (Fig. 4). The results showed that the *slc4* genes in *T. dalaica* had the highest collinearity with the zebrafish genes, and that they shared a conserved genomic neighborhood with zebrafish and Nile tilapia, confirming the reliability of the *slc4* genes annotation. Therefore, our findings not only well support the annotation of the *slc4* genes in *T. dalaica*, but again show that the *slc4* genes have been highly conserved during evolution.

**Fig. 5:**
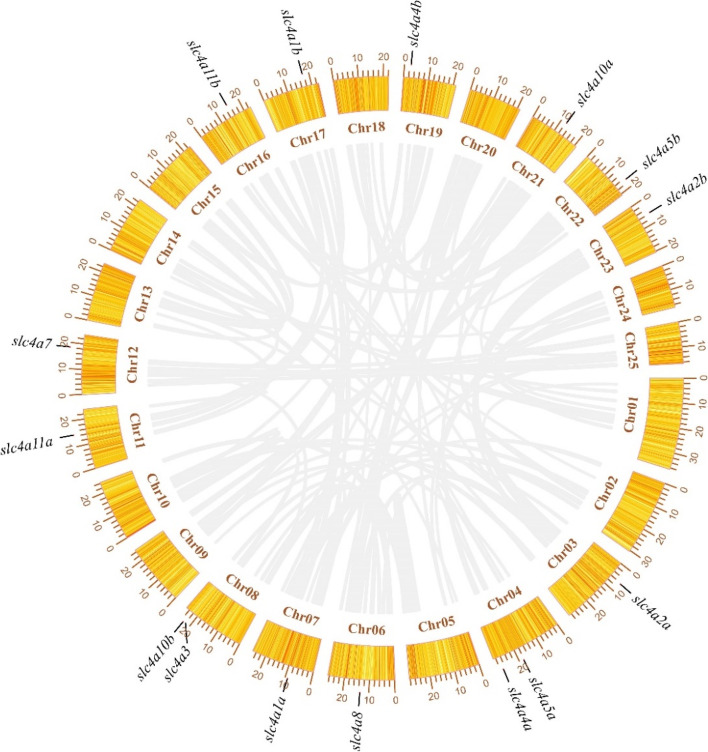
Location of the *slc4* genes on the chromosome of *T. dalaica*

In addition, this study found that the 15 *slc4* genes of *T. dalaica* were distributed on 13 chromosomes (Fig. 5). Two of the *slc4* genes (*slc4a5b, slc4a4a*) were located on chromosome 4; *slc4a10b* and *slc4a3* were located on chromosome 8, and the other 11 genes were located on different chromosomes. Compared with higher vertebrates, gene duplication is more common in teleost fishes. Therefore, based on the chromosomal collinearity analysis and the location information of each *slc4* gene, we speculate that the duplicated *slc4* genes in *T. dalaica* were primarily due to a teleost-specific whole genome duplication (WGD) event [22].

### Recombinant analysis of *slc4* family genes

Genetic recombination plays an important role in the evolution of gene families [23]. Only one gene had a recombination signal (Table 3). *Slc4a2a* has a recombination site with *slc4a1b* at 2554 ~ 2892 bp. Therefore, in order to adapt to environmental changes, *T. dalaica* increased the number of *slc4* genes and the expression of *slc4* genes through intragene recombination.

**Table 3:** The predicted recombination events for the *slc4* gene family

Detection Methods	Recombination event		
	sequence	*P* value	Breakpoint Positions
GENECONV	*slc4a2a*	3.781 × 10^−02^	2554 ~ 2892

### Analysis of the expression results of *slc4* genes

**Fig. 6:**
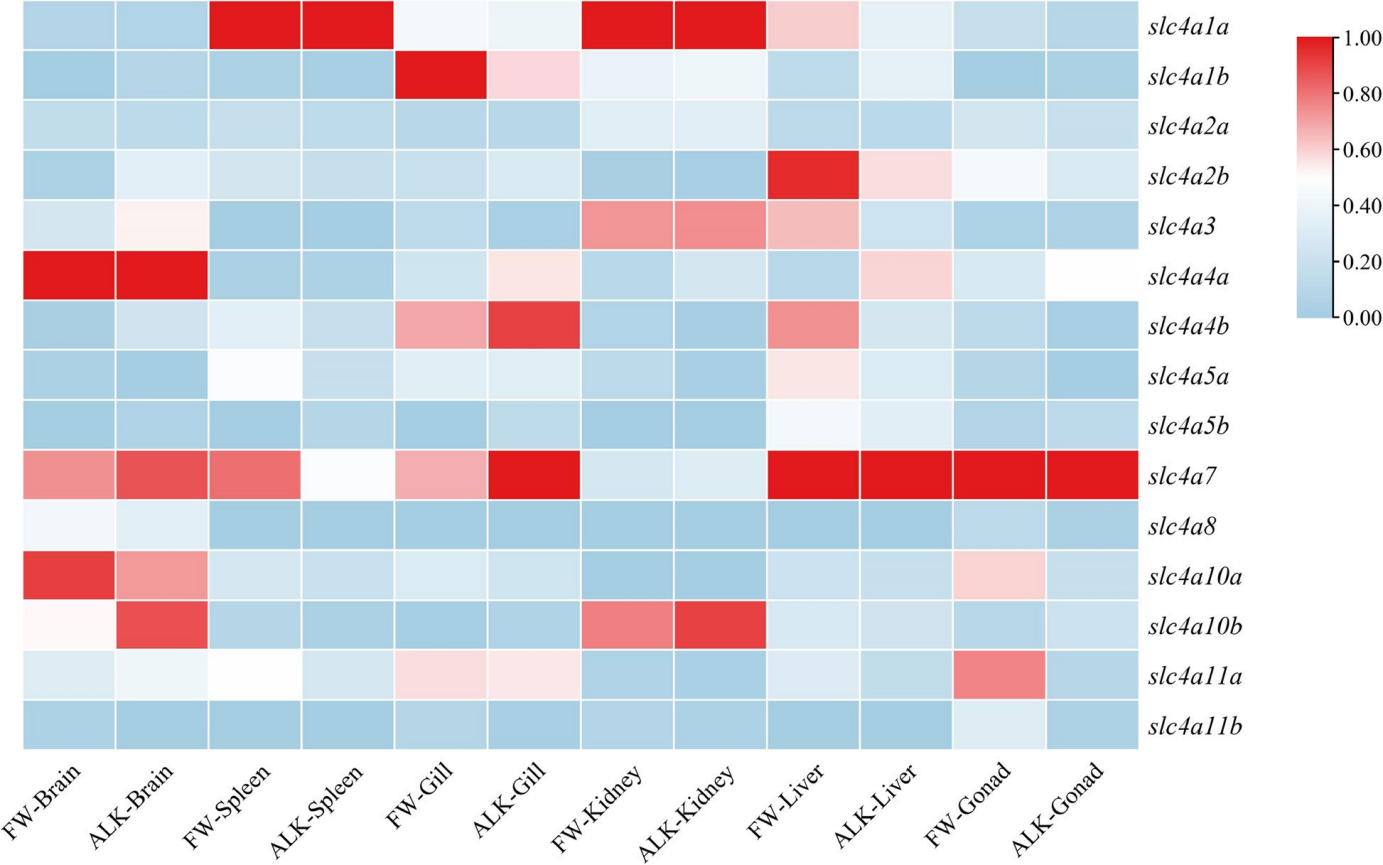
Expression of *slc4* genes in *T. dalaica* in various tissues in different habitats

Quantitative polymerase chain reaction (qPCR) was used to analyze the expression of *slc4* genes in different tissues (Fig. 6, Supplementary Fig. S1). The differential expression of *slc4* genes and the regulation mode of adapting to high salinity environments were visually displayed. In different habitats, the coping mechanisms of *slc4* genes differ, and thus their expression levels may be different. The *slc4* genes show tissue-specific expression patterns, and they play major regulatory roles in the brain, liver, gill, kidney, and other tissues. Differential expression patterns of *slc4* genes have been observed in different tissues in freshwater and alkaline water habitats. For example, *slc4a4a*, *slc4a7*, and *slc4a10a* are highly expressed in brain tissue, but are expressed at lower levels in kidney. For osmoregulatory organs, *slc4a1b, slc4a7*, and *slc4a4b* were highly expressed in the gills. In the kidney, *slc4a1a, slc4a3*, and *slc4a10b* were highly expressed. Different copies of the same gene also showed different expression patterns in different tissues. For example, relative to the freshwater group, *slc4a1a* was only highly expressed in the spleen and kidney, while *slc4a1b* was expressed at a low level in these two tissues. This indicates that the two *slc4a1* gene copies have different physiological functions in *T. dalaica*. From freshwater to alkaline water environments, some genes were upregulated, and others were downregulated. For example, *slc4a4b* was highly expressed in the liver in freshwater environments, but downregulated in alkaline water environments. *Slc4a10b* was upregulated in the brain, indicating that it has an important regulatory role in adaptation to highly alkaline environments.

## Discussion

### Characteristics of *slc4* gene family members of *T. dalaica*

The *slc4* gene family members are membrane transporters, and previous studies have shown that this gene family is involved in the transport of HCO_3_^−^ in mammals [24, 25]. In recent years, studies have shown that the *slc4* genes also play important roles in teleost fishes, and findings in zebrafish, *Leuciscus waleckii*, and *Oreochromis niloticus* suggest that some genes are involved in ion osmosis and intracellular physiological processes such as pH regulation [10–12, 14]. At present, there are few systematic studies on the entire *slc4* gene family. To have an overall understanding of the functions of the *slc4* gene family, the members of the family in *T. dalaica* were systematically analyzed by combining bioinformatics technology and real-time PCR.

In this study, a total of 15 *slc4* genes were identified in the *T. dalaica* genome distributed on 13 chromosomes, and there was no tandem duplication of the *slc4* gene copies (Fig. 5). According to the existing research, the gene family members are divided into three different branches according to their functions in ion transport. By aligning the amino acid sequences of multiple species and constructing a phylogenetic tree (Fig. 3), we found that the 15 sequences were divided into 10 branches (*slc4a1, slc4a2, slc4a3, slc4a4, slc4a5, slc4a7, slc4a8, slc4a9, slc4a10,* and *slc4a11*). Orthologous *slc4* genes from all species clustered together. The corresponding *slc4* genes of *T. dalaica* were first clustered with three species of *Triplophysa*; second, the sequences clustered with those of zebrafish. Combined with the results of the collinearity comparison of the adjacent genes of *T. dalaica*, zebrafish, and Nile tilapia (Fig. 4), this demonstrates that the *slc4* gene family is relatively conserved. It is worth noting that the *slc4* genes differ in the evolution of higher vertebrates and teleost fishes, and the phenomenon of gene deletion has been accompanied by whole genome duplication [26]. This phenomenon may be due to gene loss during evolution and may reflect ecological adaptations in teleosts and other vertebrates [27, 28].

### Expression patterns of members of the *slc4* family in *T. dalaica*

Although there are structural similarities among the *slc4* gene family members, there are different expression patterns in different tissues. In this study, qPCR showed that the *slc4* genes were expressed in the brain, spleen, gill, liver, gonad, and kidney of *T. dalaica*. *T. dalaica* from Anyang and Inner Mongolia showed differences in their ability to regulate carbonate-alkaline metabolism due to long-term adaptability in water environments with different alkalinities. The above differences were reflected in the following aspects:

1. Cl^−^/HCO_3_^−^ exchangers

Five members identified in this study (*slc4a1a, slc4a1b, slc4a2a, slc4a2b*, and *slc4a3*) belong to Cl^−^/HCO_3_^−^ exchangers that exchange monovalent anions from opposite sides of the cell membrane. The gene *slc4a1*, a Cl^−^/HCO_3_^−^ exchanger for erythrocytes, was one of the earliest identified transporters among the members of the *slc4* gene family. Guizouarn et al. found through experiments that *slc4a1* exhibits different transport characteristics in response to different stimuli [29]. *Slc4a1a* mediates Cl^−^ and HCO_3_^−^ exchange in the kidney, and it is also required for normal acidification of urine. Studies have shown that the expression level of *slc4a1b* in zebrafish gills is lower in high Na^+^ environments than in low Na^+^ environments, but higher in acidic water than in freshwater environments [21]. Shmukler et al. found that the expression levels of *slc4a2* genes were distinct in different developmental stages and tissues of the zebrafish [9]. Previous studies have found that *slc4a3* exists in mammalian excitable tissues such as brain [30, 31], heart [32, 33], and epithelial cells of the kidney and gastrointestinal tract. There is currently evidence that the zebrafish *slc4a3* gene is sensitive to pH changes; at a pH of 8.5, the anion exchange activity is significantly increased [34]. It has been hypothesized that *slc4a3* regulates pH by exporting HCO_3_^−^ upon intracellular alkali loading.

In our study, the *slc4a1a* gene expression in the kidney and spleen of *T. dalaica* in a saline-alkali fish was significantly higher than that in freshwater individuals. In *T. dalaica*, the expression of *slc4a1b* in the gill was much lower in the saline-alkali environment than in the freshwater environment, and the expression of *slc4a1b* gene in the brain, liver, and gonad was significantly increased in the saline-alkali environment. The expression patterns of *slc4a2* genes in different tissues of the two habitats were basically the same as that of *slc4a1*. *Slc4a3* was expressed to varying degrees in the brain and gonads. However, the role of *slc4a3* in mediating anion exchange in teleost development and physiology remains to be elucidated [34].

(2) Na^+^/HCO_3_^−^ cotransporters

Members of this branch transport Na^+^ and HCO_3_^−^ from one side of the cell membrane to the other [5]. According to whether the protein is electrogenic during transmembrane transport, these genes can be divided into two categories, one being electrogenic (*slc4a4* and *slc4a5*)*,* the other being electrically neutral, including *slc4a7, slc4a8*, and *slc4a10* [35].

*Slc4a4* genes are essential for the acid-base balance of various tissues of the body. Previous studies have shown that *slc4a4* genes are highly expressed in the gills, kidneys, intestines, and livers of freshwater fishes such as *Oncorhynchus mykiss* [36–38]. *Slc4a4* genes are responsible for pH regulation in the liver, whereas in the kidney, *slc4a4* genes play key roles in the reabsorption of HCO_3_^−^. Moreover, the *slc4a4* genes are also expressed in gills of *Anguilla japonica* [39] and the intestines of *Takifugu obscurus* [13] during seawater-acclimating processes. The latter study found that *slc4a4* gene expression was detected in the retina, pronephros, and gills of zebrafish. The gill has a mechanism to prevent metabolic acidosis and alkalosis. *Slc4a4* gene expression in zebrafish gills is consistent with its function observed in freshwater and marine fishes, where it is responsible for regulating osmotic pressure and pH in biological corpora [40]. It has been speculated that the systemic acid-base balance of fish is likely to be accomplished by the joint action of kidneys and gills. More studies have found that *slc4a4* gene is widely expressed in all cells in the zebrafish ventricle, and it has been speculated that it may be involved in absorption from the cerebrospinal fluid to the brain parenchyma [41, 42]. Similarly, *slc4a8* genes were found to contribute to cerebrospinal fluid secretion in mice [43], and human *slc4a8* mRNA exists in multiple parts of the human brain, testis, kidney, and ovary. The mRNA encoding *slc4a10* genes was originally found in rat brain, pituitary, testis, kidney, and ileum [44].

Our findings are largely consistent with those of previous studies. The expression of *slc4a4* genes in the gill, liver, and brain of *T. dalaica* in saline-alkali waters was significantly increased, indicating that the fish maintain ion homeostasis and acid-base balance in the body in response to a strong saline-alkali environment. The expression levels of these genes were significantly enhanced to prevent alkalosis [45]. At present, there are almost no reports or studies on *slc4a5* and *slc4a7* in teleosts. We speculate that the *slc4a7* genes may play a greater role in gill tissue, because the expression of these genes was significantly increased in saline-alkali waters. In addition, the *slc4a7* gene was also expressed in the brain, liver, and gonad tissues. Therefore, further research is needed on the expression patterns of *slc4a5* and *slc4a7* involved in acid-base regulation in vivo and their corresponding functions [46]. When fish were exposed to high alkalinity, *slc4a10b* was expressed at higher levels in the brain than in freshwater environments. In addition, the *slc4a10* genes were significantly differentially expressed in the brain, kidney, and gonad tissues of *T. dalaica* under saline-alkali stress, indicating that there may have been functional differentiation.

(3) Na^+^/Borate cotransporters

The Na^+^/Borate cotransporter is a mixed cotransporter. The Na^+^/Borate cotransporter can transport Na^+^ and two HCO_3_^−^, one Na^+^ and one CO_3_^2−^, or one NaCO_3_^−^ ion pair into the cell while exchanging one Cl^−^. In the presence of borate, *slc4a11* genes are voltage-regulated Na^+^/Borate transporters, whereas in the absence of borate, *slc4a11* genes mediate Na^+^ and OH^−^(H^+^) transport [7]. The current study found that *slc4a11* genes were required for renal urine concentration, but the link between the Na^+^/Borate cotransporter in animals and humans is unclear [47]. Our results suggest that in the brain, gills, and gonads, *slc4a11* in *T. dalaica* may be involved in the regulation of ion and acid-base/nitrogen homeostasis.

The *T. dalaica* in Dali Lake have a strong alkali tolerance due to long-term habitation of water with high carbonate alkalinity. The *T. dalaica* in Anyang city can also live normally in fresh water. This indicates that the fish have evolved multiple regulatory modes under different alkalinity environments, suggesting a potential breeding object in saline-alkali areas for aquaculture. The *slc4* genes of *T. dalaica* are upregulated or downregulated by environmental salinity to maintain survival, growth, and reproduction, and thus form a characteristic of this species to adapt to different environmental conditions. At present, this gene family has been studied in mammals, and the expression patterns of these transporters in teleost fishes have a certain similarity with those of mammals.

## Conclusions

In this study, 15 *slc4* genes were identified in the *T. dalaica* genome, and their basic information of sequence features, conserved domains, and conserved motifs were statistically analyzed. The 15 *slc4* genes were distributed on 13 chromosomes. A phylogenetic tree was established using the amino acid sequences of the *slc4* genes of representative vertebrates, and the tree was divided into three categories according to the different transport ions. We discussed the evolutionary relationships of the *slc4* genes in *T. dalaica* and found that duplicate copies of the *slc4* genes were present in common teleost genomes due to the WGD event in teleosts. The results show that the *slc4* gene family expanded significantly during the evolution of teleost fishes. The results of a collinearity analysis indicated that the *slc4* genes in *T. dalaica* had strong collinearity with those of zebrafish, and the *slc4* genes were highly conserved in the evolutionary process. This finding further supports the annotation results for the *T. dalaica* genome. The differential expression of *slc4* gene family members in six tissues including gill, kidney, and brain under different habitats was analyzed by RT-PCR. We speculated that *slc4a1, slc4a4*, and *slc4a7* genes may play greater roles in the gill. This study will help us to understand the expression patterns of *slc4* gene family members in saline-alkali environments and will provide support for subsequent studies on how *slc4* genes regulate fish adaptation to saline-alkali environments. This will aid the search for suitable breeding varieties in saline-alkali areas for aquaculture.

## Materials and methods

### Identification of *slc4* gene family members

The CDS sequences and amino acid sequences of the zebrafish *slc4* genes were obtained from the Ensembl (http://asia.ensembl.org) database, and the protein sequences of 14 zebrafish *slc4* genes were used as the query sequences. BLAST (1e-5) was performed in the genome database of *T. dalaica*, and the National Genomics Data Center under Accession number PRJNA624716. The extracted candidate genes of *T. dalaica* were subjected to reverse blast at NCBI to verify the accuracy.

### *Slc4* genes structure analysis

Based on the amino acid sequences of the *slc4* genes of *T. dalaica*, the SMART 7.0 (http://smart.embl-heidelberg.de/) tool was used to predict the conserved domain structures of the *slc4* genes, and the results were further confirmed by combining with the conserved domain prediction in NCBI. ExPASy (https://www.expasy.org/) was used to predict the isoelectric point and molecular weight of each *slc4* protein. Cell-PLoc2.0 [48] (http://www.csbio.sjtu.edu.cn/bioinf/Cell-PLoc-2/) was used to predict the subcellular localization of *slc4* gene products. The location information of the *slc4* genes and the structural information of exons and introns were obtained from the annotation file of the *T. dalaica* reference genome (GFF) and visualized using TBtools [49] software. The motifs of the identified *slc4* proteins were predicted using the MEME (http://meme-suite.org/) program. The maximum value of the searched motifs was set to 10; the minimum width was set to 6, and the maximum value was 50. The results were visualized in TBtools [49].

### Phylogenetic analysis of *slc4* genes

To further understand the phylogenetic relationships and nomenclature of the *slc4* genes, the CDS sequences and amino acid sequences of some representative species’ *slc4* genes were downloaded from the NCBI and Ensembl databases. These species were the zebrafish (*Danio rerio*), human (*Homo sapiens*), mouse (*Mus musculus*), clawed frog (*Xenopus laevis*), stickleback (*Gasterosteus aculeatus*), European sea bass (*Dicentrarchus labrax*), channel catfish (*Ictalurus punctatus*), large yellow croaker (*Larimichthys crocea*), Nile tilapia (*Oreochromis niloticus*), Japanese medaka (*Oryzias latipes*), and fugu (*Takifugu rubripes*). The genomes of these species have been well annotated. The published genome data of three species of *Triplophysa* were downloaded from the Ensembl database and Gigabase; these were *Triplophysa siluroides, Triplophysa tibetana*, and *Triplophysa bleekeri*. The identification of the *slc4* family members of these three species was similar to that for *Triplophysa dalaica*. Using the *slc4* protein sequences of zebrafish as the query sequences, the three candidate *slc4* genes of *Triplophysa* were searched by BLASTP [49] and then combined with NCBI to perform a reverse blast to obtain the final confirmed *slc4* genes.

The amino acid sequences of 202 *slc4* genes of 15 species obtained above were aligned by ClustalW2 [50] (http://www.ebi.ac.uk/Tools/msa/clustalw2/) using the default parameters. Based on the neighbor-joining method, the aligned sequences were employed in MEGA 7.0 [51] to construct a phylogenetic tree, and the Jones-Taylor-Thornton (JTT) model was selected with a bootstrap value of 1000. The evolutionary tree was further polished via the iTOL (https://itol.embl.de/itol.cgi) online site.

### Collinearity analysis and recombination events detection in *slc4* genes

Collinear analysis was conducted to compare the neighbored genetic regions of *slc4* genes among zebrafish, Nile tilapia, and *T. dalaica*. The chromosomal locations of zebrafish and Nile tilapia *slc4* family members and their adjacent genes were identified using Genomicus 97.01 (https://www.genomicus.biologie.ens.fr/genomicus-97.01/cgi-bin/search.pl). The locations of members of the *slc4* family in *T. dalaica* were derived from the results of the genome assembly annotations. The three species were mapped for collinearity based on the obtained genetic information. To further understand the evolutionary feature of *slc4* genes in the *T. dalaica.* Potential recombination events were identified using RDP v4.8 [52]. GENECONV [53] were used to analyze the sequences with 0.05 *P* value cutoff and 100 permutation parameters in the study.

### Acquisition of experimental fish

The experimental *T. dalaica* used in this study came from two habitats, Lushui River (fresh water) in Linzhou, Anyang (35°54′28.0″N, 113°51′26.0″E), and Dali Lake (saline-alkali water) in Inner Mongolia. Three samples were taken from each group. Each animal was euthanized with buffered tricaine methanesulfonate (MS-222). The six tissues of brain, liver, spleen, gonad, kidney, and gill were taken, and the tissues were placed in 400 μL RNAiso Plus (Trizol, Takara) in 1.5 ml enzyme-free centrifuge tubes. After being kept at 4 °C for 4 h, samples were stored in a − 80 °C refrigerator.

### Total RNA extraction, cDNA synthesis, and quantitative real-time PCR (qPCR) analysis

The RNA extraction method referred to the instructions of tissue preservation reagent RNAiso Plus. Extracted RNA was tested for concentration and integrity. Reverse transcription of qualified RNA was performed for each sample following the steps in the HiScript II Reverse Transcriptase manual. Primer 5.0 was used to design specific primers for the members of the *T. dalaica slc4* gene family (Table S1). The expression of *slc4* genes of *T. dalaica* in different habitats and different tissues was detected by qRT-PCR. The qPCR was conducted according to the instructions for ChamQ Universal SYBR qPCR Master Mix reagent. The total reaction volume was 10 μl, comprising 5 μl of 2 × ChamQ Universal SYBR qPCR Master Mix, 0.2 μl of forward primer, 0.2 μl of the reverse primer, 1 μl template cDNA, and 3.6 μl ddH_2_O. The reaction conditions were as follows: pre-denaturation at 95 °C for 30 s followed by 40 cycles of 95 °C for 10 s, 60 °C for 30 s, and a final extension at 72 °C for 2 min. The freshwater habitat group was used as the control group; the saline-alkali water group was used as the treatment group, and *β-actin* was used as the internal reference gene. The method of 2^-ΔΔCt^ [54] was used for data processing, and the relative expression levels were calculated. Data were normalized in TBtools [48] and the results were visualized as heatmaps to compare the expression of each gene in different tissues. The relative expression of *slc4* gene family in the two habitats was statistically graphed in the study.

## Supplementary Information

**Additional file 1: Fig. S1.** Statistically analyzed the expression of *slc4* gene family members in different tissues and habitats.

**Additional file 2: Table S1.** Primers used for qRT-PCR of *slc4* gene family.

**Additional file 3: Table S3.** Abbreviations of gene names used in synteny analysis.

## Data Availability

The datasets supporting the results of this article are included within manuscript and available on request (Dr. Chuanjiang Zhou). The *Triplophysa dalaica* genome DNA sequencing data have been deposited into the NCBI Sequence Read Archive under BioProject: PRJNA624716 (https://academic.oup.com/gbe/article/13/8/evab153/6311268). The datasets generated and analysed during the current study are available in the NCBI GenBank: OP326736- OP326750 (Data will be released when the manuscript published).
