# Supplementary figures and images for: Genome-wide characterization of the Triplophysa dalaica slc4 gene family and expression profiles in response to salinity changes
Source: BMC Genomics. 2022 Dec 13;23:824. doi: 10.1186/s12864-022-09057-8 (PMC9746111; doi:10.1186/s12864-022-09057-8)

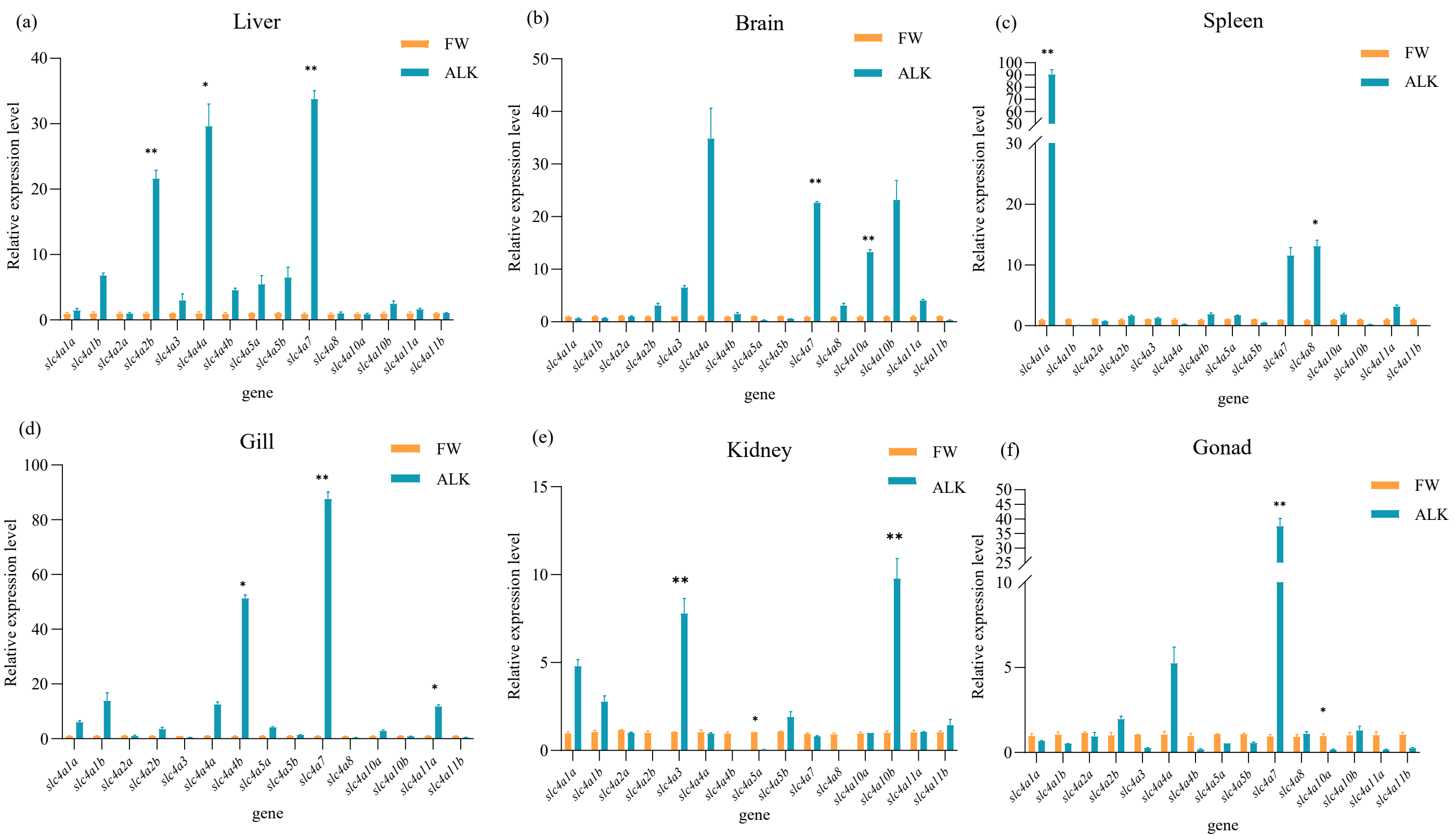

Supplement: Supplementary file 1 — Additional file 1: Fig. S1. Statistically analyzed the expression of slc4 gene family members in different tissues and habitats. [file 12864_2022_9057_MOESM1_ESM.pdf]
