# Supplementary material for: Genome-wide characterization of the Triplophysa dalaica slc4 gene family and expression profiles in response to salinity changes
Source: BMC Genomics. 2022 Dec 13;23:824. doi: 10.1186/s12864-022-09057-8 (PMC9746111; doi:10.1186/s12864-022-09057-8)
Supplement: Supplementary file 2 — Additional file 2: Table S1. Primers used for qRT-PCR of slc4 gene family. [file 12864_2022_9057_MOESM2_ESM.docx]

**Supplementary Table S1** Primers used for qRT-PCR of *slc4* gene family

| Gene | Forward primers (5'-3') | Reverse primerS（5'-3'） |
| --- | --- | --- |
| *β-actain* | AAGCCGGATTTGCTGGAGA | CGATGGGGTATTTCAGGGTCA |
| *slc4a1a* | GACTGCTGGCGGATAAGACG | GGCTGAGCGGCAAACAAA |
| *slc4a1b* | AAGGCTAAGGGCATCGAGTACA | GAGTGAATCGGGAAATGAAACG |
| *slc4a2a* | CGAAAGCTGCCAATCCTACC | GAACTCATCGGAAACAAACACG |
| *slc4a2b* | TGTTCACGGCGGTTCAAGT | AAGATGCGAGCCAGGACGA |
| *slc4a3* | CGTGGTCTTAGTGGGTTGCG | GCGTCCAATCTCGTGGTAGTC |
| *slc4a4a* | ACTGTGGCTGCTATCATCTTTCC | CACTGTCTACGCTCCCTTGTTTC |
| *slc4a4b* | ACCTGGGCACCGTAACTAATG | AACGCCGTACCAAGAAAACTC |
| *slc4a5a* | TAGCGTATAAAGCCAGGGACC | CTTTTGTCAGCCGAGGGAGT |
| *slc4a5b* | ATCCGTCTACATTGGTGTTCCG | GCTCATTTGAATCCCGCTCTT |
| *slc4a7* | ATCCCTCACGACCTCTTCACA | TTGCTCCAGCGTTCACCTC |
| *slc4a8* | CTCAGCCTCCGCCTACAAA | GACAAGCGAACAAAGGCAACTA |
| *slc4a10a* | AGAATAGAGGGGAGTTTGTCGG | ACCTTGGTTGGGAAGTAGCG |
| *slc4a10b* | TTTCTGTACTGCGCCTGTATGTC | GCGAGTAAGCAATCCCAGTCAT |
| *slc4a11a* | TCTGTCCGACTGTGCTCTACCT | CCACTCGAAACACTGGACCTT |
| *slc4a11b* | GCTGCGAACATTCTGATAGGG | CTGCGGATGTAGTGAGTGGG |
