# Supplementary material for: Genome-wide characterization of the Triplophysa dalaica slc4 gene family and expression profiles in response to salinity changes
Source: BMC Genomics. 2022 Dec 13;23:824. doi: 10.1186/s12864-022-09057-8 (PMC9746111; doi:10.1186/s12864-022-09057-8)
Supplement: Supplementary file 3 — Additional file 3: Table S3. Abbreviations of gene names used in synteny analysis. [file 12864_2022_9057_MOESM3_ESM.docx]

**Supplementary Table S2** Abbreviations of gene names used in synteny analysis.

| **Gene abbreviation** | **Gene full name** |
| --- | --- |
| *tmub2* | transmembrane and ubiquitin-like domain containing 2 |
| *atxn7l3* | ataxin-7-like protein 3 |
| *sepw2a* | selenoprotein W2a |
| *sepw2b* | selenoprotein W2b |
| *ngfra* | tumor necrosis factor receptor superfamily member 16 |
| *mien1* | migration and invasion enhancer 1 |
| *fam117ab* | uncharacterized protein LOC100151756 |
| *fam49ba* | family with sequence similarity 49 member Ba |
| *stc2* | Stanniocalcin-2 |
| *dnajb14* | DnaJ homolog subfamily B member 14 |
| *ndufa4* | cytochrome c oxidase subunit NDUFA4 |
| *ngfrb* | nerve growth factor receptor b precursor |
| *smarcd2* | SWI/SNF-related matrix-associated actin-dependent regulator of chromatin subfamily D member 2 |
| *psmc5* | 26S protease regulatory subunit 8 |
| *asap1a* | arf-GAP with SH3 domain, ankyrin repeat and PH domain 1a |
| *ubtf* | nucleolar transcription factor |
| *ttc19* | tetratricopeptide repeat protein 19 |
| *faim2a* | fas apoptotic inhibitory molecule 2a |
| *ca2* | carbonic anhydrase II |
| *abcf2a* | ATP-binding cassette sub-family F member 2a |
| *nrbp2b* | nuclear receptor binding protein |
| *atp6v0a* | V-type proton ATPase 116 kDa subunit a |
| *egfr* | epidermal growth factor receptor |
| *alox8* | Arachidonate 8S-lipoxygenase |
| *ubxn1b* | UBX domain-containing protein 1b |
| *tmeff1* | Tomoregulin-1 |
| *lct* | Lactase-phlorizin hydrolase |
| *wdsub1* | WD repeat, SAM and U-box domain-containing protein 1 |
| *cdk9* | Cyclin-dependent kinase 9 |
| *cx43.4* | gap junction gamma-1 protein |
| *hdac1* | histone deacetylase 1 |
| *igfbp2b* | insulin-like growth factor-binding protein 2b |
| *igfbp5b* | insulin-like growth factor-binding protein 5b |
| *tns1b* | tensin-1b |
| *col5a2* | collagen type V alpha 2 chain |
| *npffr2* | neuropeptide FF receptor 2 |
| *adamts3* | A disintegrin and metalloproteinase with thrombospondin motifs 3 |
| *gc* | GC vitamin D binding |
| *dck* | Deoxycytidine kinase |
| *polk* | DNA polymerase kappa |
| *cacfd1* | Calcium channel flower homolog |
| *mob1b* | MOB kinase activator 1b |
| *tanc1* | Protein TANC1 |
| *ankdd1b* | ankyrin repeat and death domain-containing protein 1B |
| *fpgs* | Folylpolyglutamate synthase |
| *pde8b* | High affinity cAMP-specific and IBMX-insensitive 3',5'-cyclic phosphodiesterase 8B |
| *ogfod2* | 2-oxoglutarate and iron-dependent oxygenase domain-containing protein 2 |
| *tacr1a* | tachykinin receptor 1a |
| *ncaph* | condensin complex subunit 2 |
| *tspan15* | tetraspanin 15 |
| *gin1* | Gypsy retrotransposon integrase-like protein 1 |
| *vps37b* | vacuolar protein sorting-associated protein 37B |
| *cnnm4a* | Metal transporter CNNM4 |
| *abcb9* | ATP-binding cassette sub-family B member 9 |
| *tacr1b* | tachykinin receptor 1b |
| *scarb1* | scavenger receptor class B member 1 |
| *tuba7l* | Tubulin alpha 71 |
| *ubc* | polyubiquitin-C |
| *sec14l7* | SEC14-like protein 2 |
| *dpy19l1l* | probable C-mannosyltransferase DPY19L1 |
| *tbx20* | T-box transcription factor TBX20 |
| *herpud2* | homocysteine-responsive endoplasmic reticulum-resident ubiquitin-like domain member 2 protein |
| *ankrd34a* | Ankyrin repeat domain-containing protein 34a |
| *polr3rlb* | polymerase (RNA) III (DNA directed) polypeptide G like b |
| *txnipb* | thioredoxin interacting protein b |
| *ip6k2b* | Inositol hexakisphosphate kinase 2b |
| *col7a1* | Collagen alpha-1(VII) chain |
| *ccdc174* | Coiled-coil domain-containing protein 174 |
| *ghrl* | Ghrelin |
| *ect2* | Protein ECT2 |
| *mcf2l2* | MCF.2 cell line derived transforming sequence-like 2 |
| *b3gnt5a* | UDP-GlcNAc:betaGal beta-1,3-N-acetylglucosaminyltransferase 5a |
| *rbms1a* | RNA-binding motif, single-stranded-interacting protein 1a |
| *rbms1b* | RNA-binding motif, single-stranded-interacting protein 1b |
| *tgfr2* | TGF-beta receptor type-2 |
| *cdk2* | Cyclin-dependent kinase 2 |
| *prmi48* | Pim proto-oncogene, serine/threonine kinase, related 47 |
| *ttll3* | tubulin monoglycylase TTLL3 |
| *snx6* | Sorting nexin-6 |
| *psmd14* | 26S proteasome non-ATPase regulatory subunit 14 |
| *tbr1a* | T-box brain protein 1a |
| *tbr1b* | T-box brain protein 1b |
| *ifih1* | interferon-induced helicase C domain-containing protein 1 |
| *fap* | fibroblast activation protein, alpha |
| *gcgb* | glucagon precursor |
| *tank* | TRAF family member-associated NF-kappa-B activator |
| *ky* | kyphoscoliosis peptidase |
| *cfd* | complement factor D precursor |
| *rpia* | ribose-5-phosphate isomerase |
| *eif2ak3* | eukaryotic translation initiation factor 2-alpha kinase 3 |
| *atrn* | attractin |
| *foxi2* | forkhead box I2 |
| *gfra4b* | GDNF family receptor alpha-4b |
| *smyd5* | SET and MYND domain-containing protein 5 |
| *harbil* | Putative nuclease HARBI1 |
| *enox2* | ecto-NOX disulfide-thiol exchanger 2 |
